# Rational design of a laminate-structured flexible sensor for human dynamic plantar pressure monitoring

**DOI:** 10.1038/s41378-024-00717-1

**Published:** 2024-07-16

**Authors:** Zuoping Xiong, Yuanyuan Bai, Lianhui Li, Zhen Zhou, Tie Li, Ting Zhang

**Affiliations:** 1 School of Nano-Tech and Nano-Bionics, University of Science and Technology of China, Hefei, Anhui 230026 P. R. China; 2 i-lab, Nano-X Vacuum Interconnected Workstation, Suzhou Institute of Nano-Tech & Nano-Bionics (SINANO), Chinese Academy of Sciences (CAS), Suzhou, Jiangsu 215123 P. R. China; 3 Suzhou Leanstar Electronic Technology Co., Ltd, Suzhou, Jiangsu 215000 P. R. China

**Keywords:** Electrical and electronic engineering, Nanosensors

## Abstract

Flexible sensors are essential components in emerging fields such as epidermal electronics, biomedicine, and human-computer interactions, and creating high-performance sensors through simple structural design for practical applications is increasingly needed. Presently, challenges still exist in establishing efficient models of flexible piezoresistive pressure sensors to predict the design required for achieving target performance. This work establishes a theoretical model of a flexible pressure sensor with a simple laminated and enclosed structure. In the modeling, the electrical constriction effect is innovatively introduced to explain the sensitization mechanism of the laminated structure to a broad range of pressures and to predict the sensor performance. The experimental results confirmed the effectiveness of the theoretical model. The sensor exhibited excellent stability for up to three million cycles and superior durability when exposed to salt solution owing to its simple laminated and enclosed structural design. Finally, a wearable sensing system for real-time collection and analysis of plantar pressure is constructed for exercise and rehabilitation monitoring applications. This work aims to provide theoretical guidance for the rapid design and construction of flexible pressure sensors with target performance for practical applications.

## Introduction

Pressure sensors are widely used in various industrial control environments, including smart cities, smart homes, production control, aerospace, and many other industries^1–3^. Currently, commonly used commercial pressure sensors are mainly based on semiconductor and microelectromechanical system (MEMS) technology^4–6^, which are characterized by miniaturization, high accuracy, and good reliability. However, the rigidity of semiconductors makes it difficult for MEMS pressure sensors to adapt to complex curved and flexible surfaces, limiting their applications in emerging fields such as epidermal electronics, biomedicine, and human-computer interaction. With the development of nanotechnology and flexible electronic technology, new types of sensors based on flexible substrates, namely, flexible sensors^7^, have emerged. These sensors exhibit the characteristics of large area, lightweight, and flexibility and have become a research hotspot in recent years^8–15^.

To meet the various demands in practical applications, the rational design of pressure sensors is highly important, and many unique structures have been designed for flexible pressure sensors to achieve specific requirements, such as high sensitivity. For example, Bao et al. designed pyramidal microstructures with optimal compressibility for flexible capacitive pressure sensors to achieve high sensitivity^16^. Similarly, Yang et al. investigated the sensitivity and linearity of flexible capacitive pressure sensors with different microstructures^17^. Other variations, such as porous structures, biomimetic structures, and serpentine structures have also been reported^18–20^. Commonly, these microstructures are achieved through micro/nanofabrication of flexible substrates. However, it is difficult for these microstructured substrates to achieve high stability during periodic use, and large-scale production is currently not possible for flexible sensors based on microstructural design. Therefore, it is particularly important to achieve high-performance sensors through simple structural design for practical applications. Finite element modeling has proven to be an efficient approach for prediction prior to the design required for creating specialized and high-performance sensors^21^. For commercialized MEMS sensors, finite element models of the sensors have been well established for rational design. However, for emerging flexible pressure sensors, challenges still exist in establishing efficient models for designing flexible pressure sensors with targeted performance, especially for flexible piezoresistive pressure sensors, which are among the most widely used types of flexible pressure sensors.

In this paper, a finite element model that innovatively introduces the electrical constriction effect is established for the theoretical calculation and prediction of the performance of a flexible pressure sensor with a simple laminate structure. The sensitization tuning mechanism of the laminate-structured sensor to a broad range of pressures is revealed, which provides theoretical guidance for the design and construction of flexible pressure sensors for practical applications. A laminate-structured flexible pressure sensor was fabricated, and the effectiveness of the model was experimentally confirmed. Flexible pressure sensors exhibit unique advantages for practical applications, with excellent stability for up to 3 million cycles and superior durability in salt solutions owing to their simple laminated and enclosed structure. Finally, a wearable system based on laminate-structured flexible pressure sensor arrays was constructed, achieving real-time collection and analysis of plantar pressure distribution for exercise and rehabilitation monitoring applications.

## Results and discussion

### The electrical constriction effect

**Fig. 1:**
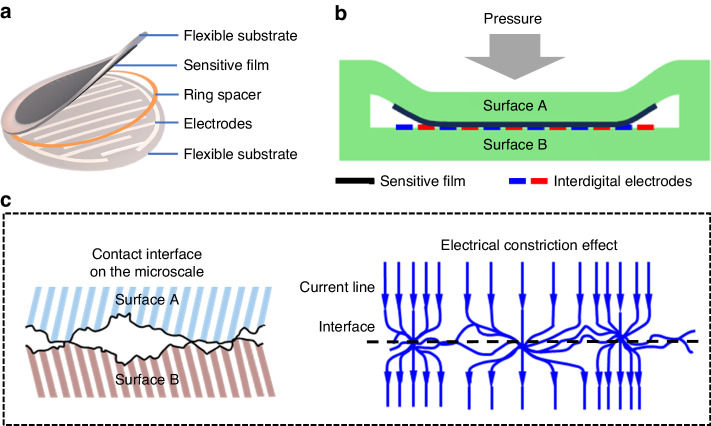
**Schematics showing the design of a laminate-structured flexible pressure sensor.** **a**, **b** Structural diagrams of the sensor. **c** The constriction of the current line at the contact interface, where the contact of two surfaces is achieved through a limited number of spots on the microscale

As illustrated in Fig. 1a, a laminate-structured flexible pressure sensor consisting of a flexible sensing layer and a flexible interdigital electrode layer separated by a ring spacer is designed. When an increasing external pressure is applied to the sensing layer (surface A), it bends toward the interdigital electrode layer (surface B) until the two surfaces touch each other (Fig. 1b), generating conductive paths at the contact interface. At this point, if a voltage potential is applied between the interdigital electrodes, an electric current will flow from one electrode through the contact interface and then to the other electrode. It is predictable that the mechanical-electrical performance of the sensor is dominated by the electrical conductivity at the contact interface. For an ideal smooth interface, the electric current flows uniformly through the contact interface, and the interfacial conductivity is nearly constant. However, solid surfaces are rough at the microscale, and contact between two surfaces is achieved through a limited number of spots. Therefore, when current flows through the contact area, it flows from a relatively large cross-sectional area to a few contact spots with small cross-sectional areas, resulting in an increased current path length (Fig. 1c)^22^. This phenomenon, known as the electrical constriction effect, has a great influence on the sensor performance, and in this paper, the electrical constriction effect is taken into account to construct a finite element model of the laminate-structured flexible pressure sensor for theoretical prediction of the sensor performance.

### Finite element modeling of the flexible pressure sensor

A finite element model of the sensor and a structural steel head for applying pressure to the sensor were established using COMSOL Multiphysics software (Supplementary Materials Figure S1). The physical fields of solid mechanics and electric currents were set up, and the main setting conditions and the geometric and material parameters can be found in the Supplementary Materials.

**Fig. 2:**
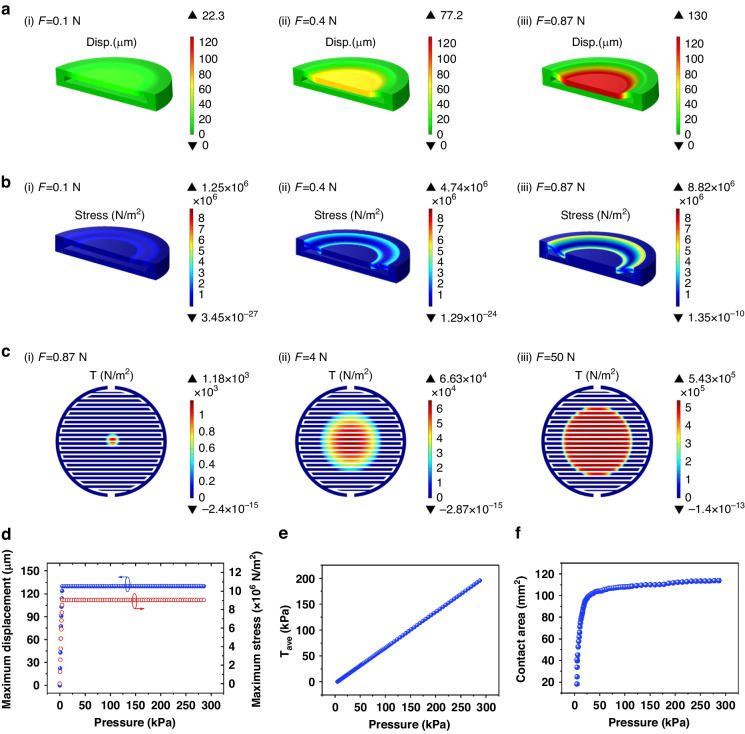
**Simulation of the mechanical response of the sensor to different pressures.** **a** Displacement (Dips.) distribution; **b** stress distribution; **c** contact pressure (*T*) distribution at the interface between the sensitive layer and the electrode; **d**–**f** variation in the calculated maximum displacement and stress of the sensor (**d**), average contact pressure (*T*_ave_) (**e**), and contact area (**f**) as a function of the applied pressure

In the physical field of solid mechanics, mechanical deformation follows the Lagrangian equilibrium equations^23,24^:

1 $${\rho }_{0}\frac{{\partial }^{2}{\bf{u}}}{\partial {t}^{2}}=\nabla \cdot {({\rm{FS}})}^{{\rm{T}}}+{{\bf{F}}}_{{\rm{V}}}$$

2 $${\rm{F}}={\rm{I}}+\nabla {\bf{u}}$$

where *ρ*_0_ is the initial mass density, **u** is the displacement, F is the deformation gradient tensor, S is the second-order Piola–Kirchhoff stress tensor, and **F**_V_ is the volume force of the sensor in the current configuration but given with respect to the undeformed volume. When the sensor reaches a steady-state under force F, $\frac{\partial {\bf{u}}}{\partial t}=0$. By solving Eqs. (1) and (2), the displacement and stress of the sensor are obtained and are presented in Fig. 2a, b and Supplementary Materials Movie S1 after data postprocessing. As the sensor is subjected to an increasing normal pressure, its upper substrate, along with the sensitive film, bends toward the electrode layer and then contacts the sensitive film. During the process, the maximum displacement of the upper substrate and the maximum stress gradually increase and then maintain a constant maximum value (Fig. 2d). The simulation results also indicate that high stress is located at the contact boundary between the gasket and the upper substrate, as well as at the contact edge between the upper substrate and the electrode; therefore, it can be inferred that mechanical failure issues such as debonding caused by excessive stress are most likely to occur at these two locations in practical use.

The strain state of the sensitive layer of the sensor is directly related to its electrical performance. By solving $\varepsilon =\frac{1}{2}[{(\nabla {\bf{u}})}^{{\rm{T}}}+\nabla {\bf{u}}+{(\nabla {\bf{u}})}^{{\rm{T}}}\nabla {\bf{u}}]$, the volumetric strain (*ε*_vol_) of the sensitive film under different applied forces is obtained and presented in Figure S2 (Supplementary Materials), which shows that the edge area of the sensitive film and the central area in contact with the electrode layer are compressed (*ε*_vol_ < 0), while other areas are stretched (*ε*_vol_ > 0). The average volumetric strain of the whole sensitive film is calculated to be greater than zero, and it sharply increases in a smaller pressure range and then decreases in a larger pressure range (Supplementary Materials Figure S3), thus reflecting that the entire sensitive film is under an equivalent tensile state over the whole pressure range.

The mechanical-electrical coupling performance of the flexible sensor is further simulated based on the simulation results of the mechanical performance of the sensor. First, an excitation voltage of 2 V is applied to the input ends of the sensor, which generates a constant 2 V potential difference between the interdigital electrodes (Supplementary Materials Figure S4a). Then, an electrical contact accompanying the mechanical contact at the interface between the sensitive layer and the electrode layer is established. Taking the electrical constriction effect into account, the interface conductivity (*σ*_*x*_) is set as follows:

3 $${\sigma }_{x}={\rm{C}}+k\,\cdot\, {T}^{{\rm{n}}}$$

where C is a constant corresponding to the interface conductivity of an ideal smooth interface. *k* · *T*^n^ is the constriction conductivity, k is a constant related to the roughness of the contact interface, the effective Young’s modulus and Poisson’s ratio of the materials, *T* is the contact pressure at the interface, and n is a constant between 0 and 1.

According to Eq. (3), the contact pressure (*T*) at the interface is closely related to the coupling electrical performance of the sensor, so its distribution and variation with increasing pressure are analyzed. As shown in Fig. 2c and Supplementary Materials Movie S2, the contact pressure starts to appear in the central area at the interface as the applied pressure increases to a critical value, with its value gradually decreasing from the center to the edge. The average *T* and its coverage area are calculated and plotted as a function of the applied pressure in Fig. 2e, f, showing that the average *T* increases linearly with increasing applied pressure, but its coverage area increases rapidly in the small-pressure range and then remains almost constant in the high-pressure range. Because the contact conductance at the interface is the integral of *σ*_*x*_ over the contact area, it could be deduced that the contact conductance will increase rapidly as the contact area increases in the small-pressure range and then increase almost linearly as the contact pressure increases in the high-pressure range.

**Fig. 3:**
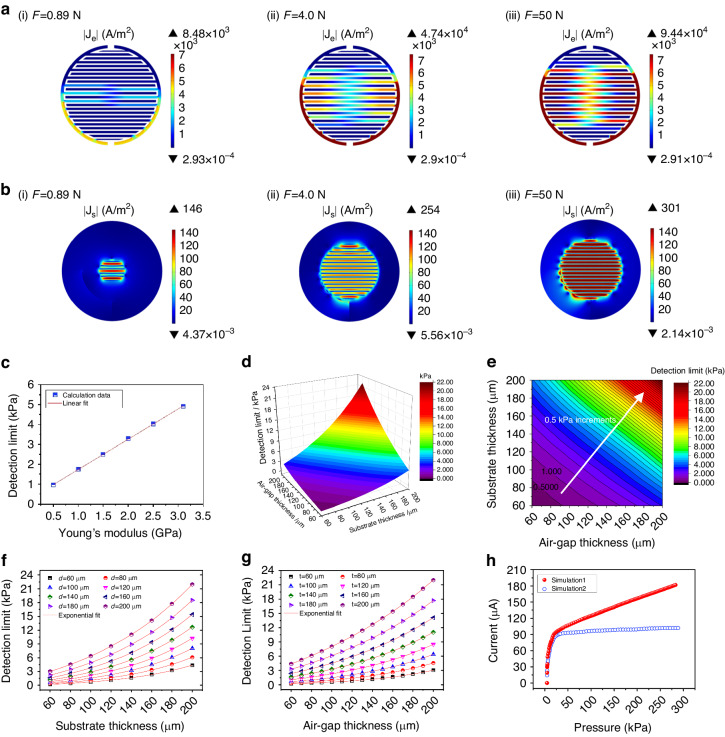
**Simulation of the mechanical-electrical coupling performance of the laminate-structured flexible pressure sensor.** **a**, **b** distribution of **a** the current density on the electrodes (|J_e_|) and **b** the sensitive layer (|J_s_|) under different applied pressures; **c** variation in the detection limit with Young’s modulus of the substrate and its fitting curve; **d** 3D color map surface; and **e** 2D color-filled contour plot showing the relationship between the detection limit and the thickness of the air gap and the substrate; **f** variations in the detection limit with the thickness of the air gap and the fitting curves at different substrate thicknesses (denoted by *t*); **g** variations in the detection limit with the thickness of the substrate and the fitting curves at different air gap thicknesses (denoted by **d**); **h** comparison of the simulation results of the mechanical-electrical coupling performance of the sensor. Simulation 1 takes the electrical constriction effect into account, while Simulation 2 does not

The steady-state conduction current density (**J**_c_) was calculated following a current conversion equation based on Ohm’s law^25–27^. As shown in Figure S4b, c (Supplementary Materials), the current density in the electrode layer is on the order of 10 S/m^2^, and that in the sensitive layer is almost zero when the applied pressure is zero. As the applied pressure increases beyond a critical value, the maximum current density in the electrode layer increases by three orders of magnitude to the order of 10^4^ S/m^2^ (Fig. 3a; the synchronous changes in the current density in the electrode layer as the applied pressure increases are presented in Supplementary Materials Movie S3), and that in the sensitive layer increases to the order of 10^2^ S/m^2^ (Fig. 3b). The critical pressure reflects the detection limit of the sensor, which is directly related to Young’s modulus of the flexible substrate as well as the thickness of the air gap and the substrate. The detection limit decreases linearly with decreasing Young’s modulus of the flexible substrates (Fig. 3c), and the relationship between the detection limit and the thickness of the air gap and the substrate is presented in Fig. 3d, e, showing that a smaller detection limit can be obtained by decreasing the thickness of the air gap or the substrate, and vice versa. Further fitting analysis verified that the detection limit decreases exponentially with decreasing thickness of either the air gap or the substrate (Fig. 3f, g; the fitting parameters are listed in Tables S1, S2 in the Supplementary Materials). In addition, the geometric design of the interdigital electrodes has no influence on the detection limit of the sensor. However, as the width of the electrode increases, the average contact pressure on the electrode decreases, but its coverage area increases, resulting in an increase in the current flowing through the sensor. When the width of the electrode does not change but the gap of the electrode increases, the average contact pressure on the electrode increases, but its coverage area decreases, resulting in a decrease in the current flowing through the sensor (Figure S5).

Figure 3a, b illustrates that the current flows from one electrode to the sensitive film and then through the contact interface to the other electrode. The total current flowing through the sensor is calculated and plotted as a function of the applied pressure in Fig. 3h, showing a sharp increase in the small-pressure range but a nearly linear increase in the high-pressure range, which could be explained by the change in the contact conductance mentioned above. As a comparison, a simulation result that does not consider the electrical constriction effect is also presented, which also shows a sharp increase in the current in the small-pressure range but a nearly constant current in the high-pressure range. The simulation results indicate that the electrical constriction effect at the rough interface between the active layer and the electrode layer is the reason why the laminate-structured sensor responds to high pressures.

### Fabrication and characterization of the flexible pressure sensor with a laminated structure

**Fig. 4:**
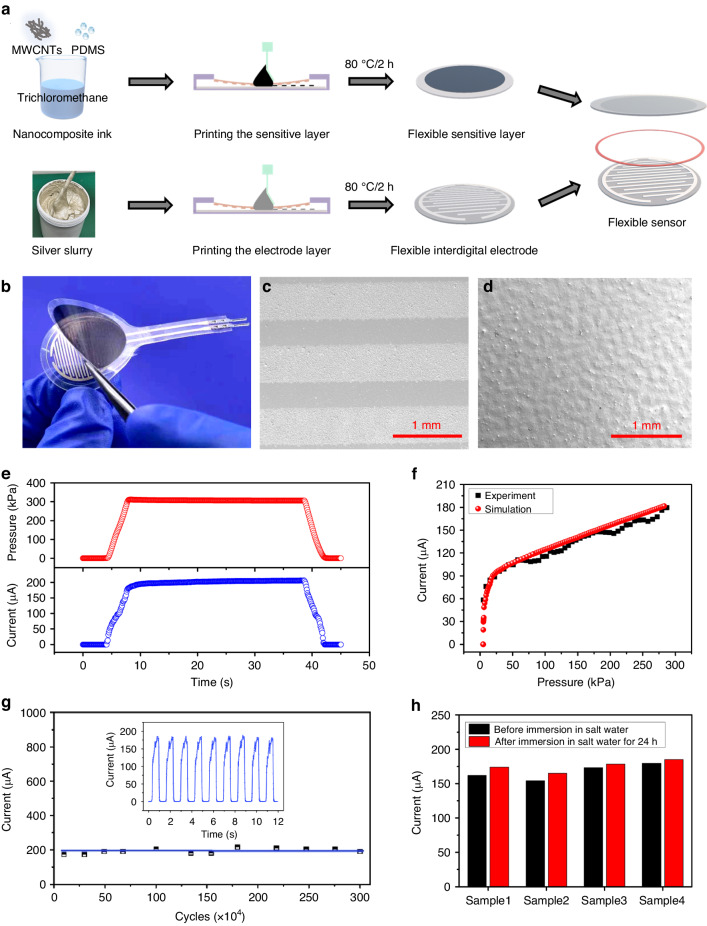
**Typical flexible pressure sensor with a laminated structure.** **a** Schematic of the fabrication process; **b** optical photograph of the sensor; **c**, **d** SEM images of the electrodes and the sensitive film of the sensor showing their micromorphologies; **e** variation in the pressure applied to the sensor with time (top) and variation in the current response of the sensor with time (bottom); **f** relationship between the current response of the sensor and the applied pressure. **g** The electrical response of the sensor within 3 million cycles (pressure of 300 kPa, 45 times/min). The inset figure depicts the real-time electrical response of the sensor, reflecting a highly stable and repeatable response of the sensor. **h** The electrical response of four sensor samples under an applied pressure of 300 kPa before and after being immersed in a sodium chloride solution for 24 h

To verify the effectiveness of the established finite element model, a laminate-structured flexible pressure was applied following the typical process shown in Fig. 4a. Generally, a nanocomposite ink containing multiwalled carbon nanotube (MWCNT) and polydimethylsiloxane (PDMS) prepolymer was screen printed onto a piece of polyethylene terephthalate (PET) substrate and thermally cured at 80 °C for 2 h to obtain a sensitive layer. By adjusting the ratio of carbon nanoparticles to the polymer substrate, the conductivity of the sensitive film was adjusted to the set value in the simulation. The interdigital electrode layer was prepared by screen printing a silver slurry onto another piece of PET substrate and thermally treating it at 100 °C for 2 h. Finally, the laminate-structured flexible sensor was achieved by bonding the sensitive layer and the electrode layer using a double-sided adhesive spacer ring (a typical photograph of the sensor is shown in Fig. 4b). Scanning electron microscopy (SEM) images of the electrodes and the sensitive film of the sensor show that both of them have rough surfaces on the microscale (Fig. 4c, d).

The electrical response of the flexible sensor to increasing external pressure applied through a structural steelhead was measured using a digital source meter. As shown in Fig. 4e, the current flowing through the sensor and the applied pressure exhibit similar synchronous changes, while the response time and relaxation time are measured to be 1 ms and 2 ms, respectively (Figure S6), which are much faster than those of most previously reported flexible pressure sensors^28,29^. Figure 4f shows that the current flowing through the sensor is very small (nA level) when the applied pressure is zero, and it does not show an apparent change until the pressure increases to a critical value, at which point it sharply increases by three orders of magnitude to the μA level. After that, the current increases rapidly in the small-pressure range and then increases linearly in the high-pressure range. In addition, the experimental results are consistent with the simulation results of the model that takes the electrical constriction effect into account.

**Fig. 5:**
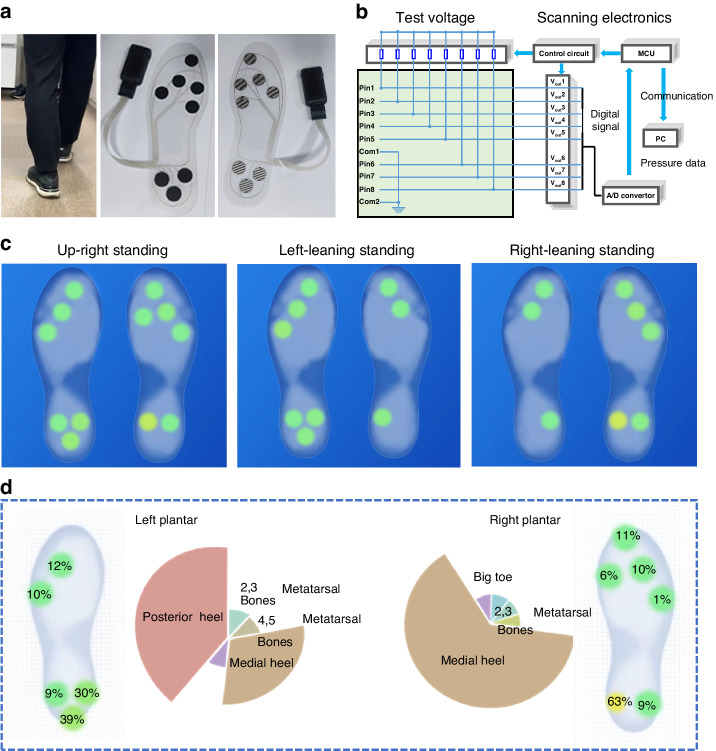
**A wearable system based on laminate-structured flexible pressure sensor arrays and wearable miniaturized circuit boards for plantar pressure mapping.** **a** Optical photographs showing the components of the system and the scenarios for monitoring the plantar pressure; **b** schematic diagram showing the collection and processing of the signals from the flexible sensor arrays; **c** plantar pressure mappings when the human body is in different standing postures; **d** pressure on different parts of the plantars under upright standing conditions of the human body

Owing to the simple laminated and enclosed structure, the response of the flexible sensor did not change during three million cycles of loading‒unloading tests (Fig. 4g) or after being immersed in a sodium chloride solution for 24 h (Fig. 4h), reflecting that it has excellent stability and durability for practical applications that are much better than those of other reported flexible pressure sensors^28^. As a demonstration, a pair of flexible, intelligent insoles composed of laminate-structured flexible pressure sensor arrays for monitoring the distribution of plantar pressure were fabricated (Fig. 5a). Plantar pressure monitoring has broad applications in the fields of human exercise and healthcare^30–32^. For example, in the field of rehabilitation, long-term collection and analysis of the plantar pressure data of patients with movement disorders during exercise can provide information on their step frequency, gait stability and symmetry, center of gravity, and exercise intensity, etc., thus allowing real-time monitoring of the rehabilitation process and enabling patients to receive more scientifically-guided rehabilitation treatment^30–32^. In principle, smaller size and greater number and density of the sensor units will lead to a higher resolution ratio in the monitoring of plantar pressure, but at the same time, the crosstalk between the leads of the sensing units will be greater, and more back-end acquisition circuit channels will be needed. When the human body is in a standing or walking state, only a few key points on the sole and heel of the foot are subjected to force, and the arch of the foot is not subjected to force. Therefore, only five and three sensing units are designed for the corresponding positions of the sole and heel on a flexible insole-shaped PET substrate, respectively, and there is no sensor designed for the arch of the foot (Fig. 5a).

To collect and process the signals from the sensor arrays, wearable miniaturized circuit boards composed of modules of the voltage divider circuit, control circuit, analog to digital (A/D) converter, and microcontroller unit (MCU) were designed and fabricated (Fig. 5a, b). In the voltage divider circuit, the flexible sensor arrays are connected in series with the voltage divider resistors. The control circuit, A/D converter and MCU are used to collect, filter, condition, and convert the voltage signals of the sensors to digital signals, which are transmitted to predeveloped software for analyzing and plotting plantar pressure. The test results indicate that the different standing postures of the human body, such as standing upright, left-leaning, and right-leaning, are directly reflected in the difference in the plantar pressure distributions (Fig. 5c). Moreover, the plantar pressure data and the degree of pressure on different parts of the plantars can also be collected, analyzed and output in real-time (Figure S7 and Fig. 5d), which is superior to most of the reported plantar pressure monitoring systems (Table S3 in the Supplementary Materials). The results also verified the feasibility of the application of as-designed plantar pressure monitoring in the fields of exercise and rehabilitation monitoring.

## Conclusion

In conclusion, a flexible pressure sensor based on a laminated structure is designed and fabricated, and a finite element model introducing the electrical constriction effect is established to investigate the working mechanism of the sensor and to evaluate the mechanical-electrical coupling performance, which provides theoretical guidance for the design and construction of flexible pressure sensors for practical applications. The electrical response of the laminate-structured flexible sensor to high pressure is attributed to the electrical constriction effect at the rough interface, which is positively correlated with the roughness of the contact interface. In addition, owing to its simple laminated and enclosed structure, the flexible pressure sensor exhibits excellent stability for up to 3 million cycles and superior durability in salt solutions for practical applications. A wearable system based on laminate-structured flexible pressure sensor arrays is finally constructed, achieving real-time collection and analysis of plantar pressure distribution for exercise and rehabilitation monitoring applications.

## Materials and methods

### Materials

Multiwalled carbon nanotubes with an average length of 10–30 μm and an average diameter of 10–20 nm (>95 wt.% purity) were purchased from Nanjing XFNANO Materials Tech Co., Ltd., China. The analytical reagent trichloromethane was obtained from Sinopharm Chemical Reagent Co., Ltd., China. The silver slurry was purchased from Shanghai Jiuyin Electronics Incorporation, China, and used as received. Polydimethylsiloxane (PDMS) was purchased from Dow Corning Corporation in Japan. PET with a thickness of 130 μm and a Young’s modulus of 3.1 GPa was purchased from DuPont Company.

### Fabrication of flexible pressure sensors with laminated structures

Carbon nanotubes were dispersed in trichloromethane with a mass ratio of 1:4 by ultrasonication for ~2 h, followed by the addition of polydimethylsiloxane prepolymer (Dow Corning Sylgard 184 with Parts A and B at 1:8 wt.%) with a mass ratio of 1:10 and mechanical stirring for ~2 h to obtain a viscous slurry. Then, the as-prepared slurry was screen printed onto a piece of PET substrate through a precustomized mask, which was thermally cured at 80 °C for 2 h to obtain the sensitive layer. Silver slurry was screen printed onto another piece of PET substrate through a precustomized mask and thermally treated at 80 °C for 2 h to form interdigital electrodes. Finally, the sensitive layer, the electrode layer, and a double-sided adhesive spacer ring were pressed together to obtain a multilayered sensor. Finally, the PINs were pressed onto the sensor for electrical performance measurements.

### Measurement and characterization of the flexible pressure sensor

SEM images of the electrode and sensitive layer of the sensor were obtained using a scanning electron microscope (FEI Quanta FEG 250) at 3 kV.

To measure the mechanical-electrical performance of the flexible pressure sensor, the sensor was placed horizontally on a pressure testing platform and fixed at the edges. The sensor was connected to a digital source meter (Keithley 2602 A) via conductive silver wires. A digital control panel along with a force gauge (Mark-10, Series 5, Force Gauge Model M5-10) was used to apply a normal force to the upper surface of the multilayered sensor through a cylindrical probe with a diameter of 15 mm. To measure the electrical response of the sensor to different pressures, the probe was kept moving at a speed of 13 mm/min to a maximum force of 50 N, and the real-time normal force and the current flowing through the sensor under a constant voltage of 2 V were recorded in synchrony every 100 ms. To measure the stability of the flexible pressure sensor, a periodic pressure of 300 kPa at a frequency of 45 times per minute was applied to the sensor, and the current was recorded synchronously. To measure the dual performance of the flexible pressure sensor, two sensor samples were immersed in a sodium chloride solution for 24 h, and their electrical responses were measured before and after immersion.

### Supplementary information

Supplementary Movie S1

Supplementary Movie S2

Supplementary Movie S3

Supporting Information
