# Supplementary figures and images for: Rational design of a laminate-structured flexible sensor for human dynamic plantar pressure monitoring
Source: Microsyst Nanoeng. 2024 Jul 16;10:98. doi: 10.1038/s41378-024-00717-1 (PMC11251139; doi:10.1038/s41378-024-00717-1)

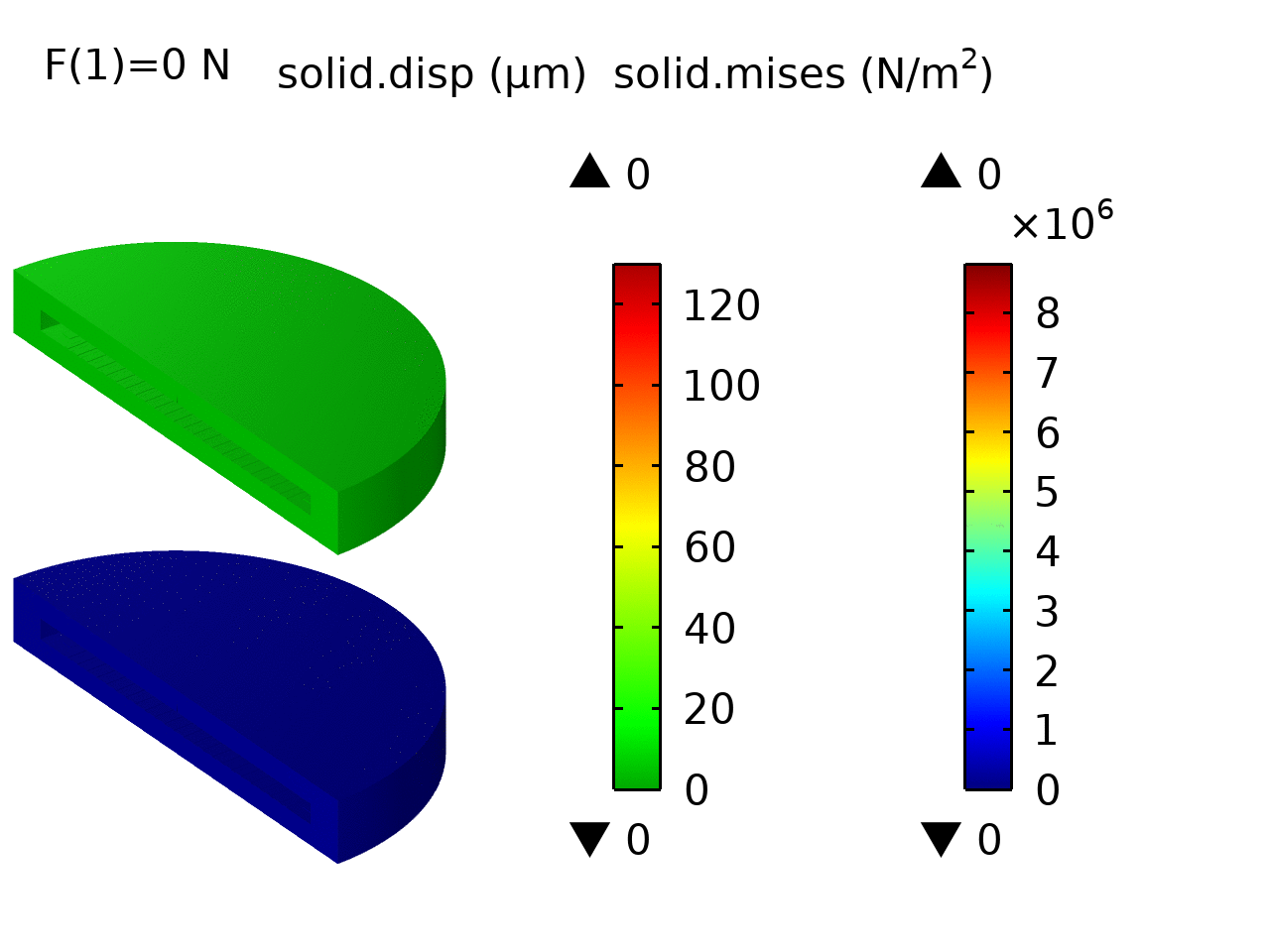

Supplement: Supplementary file 1 — Supplementary Movie S1 [file 41378_2024_717_MOESM1_ESM.gif]

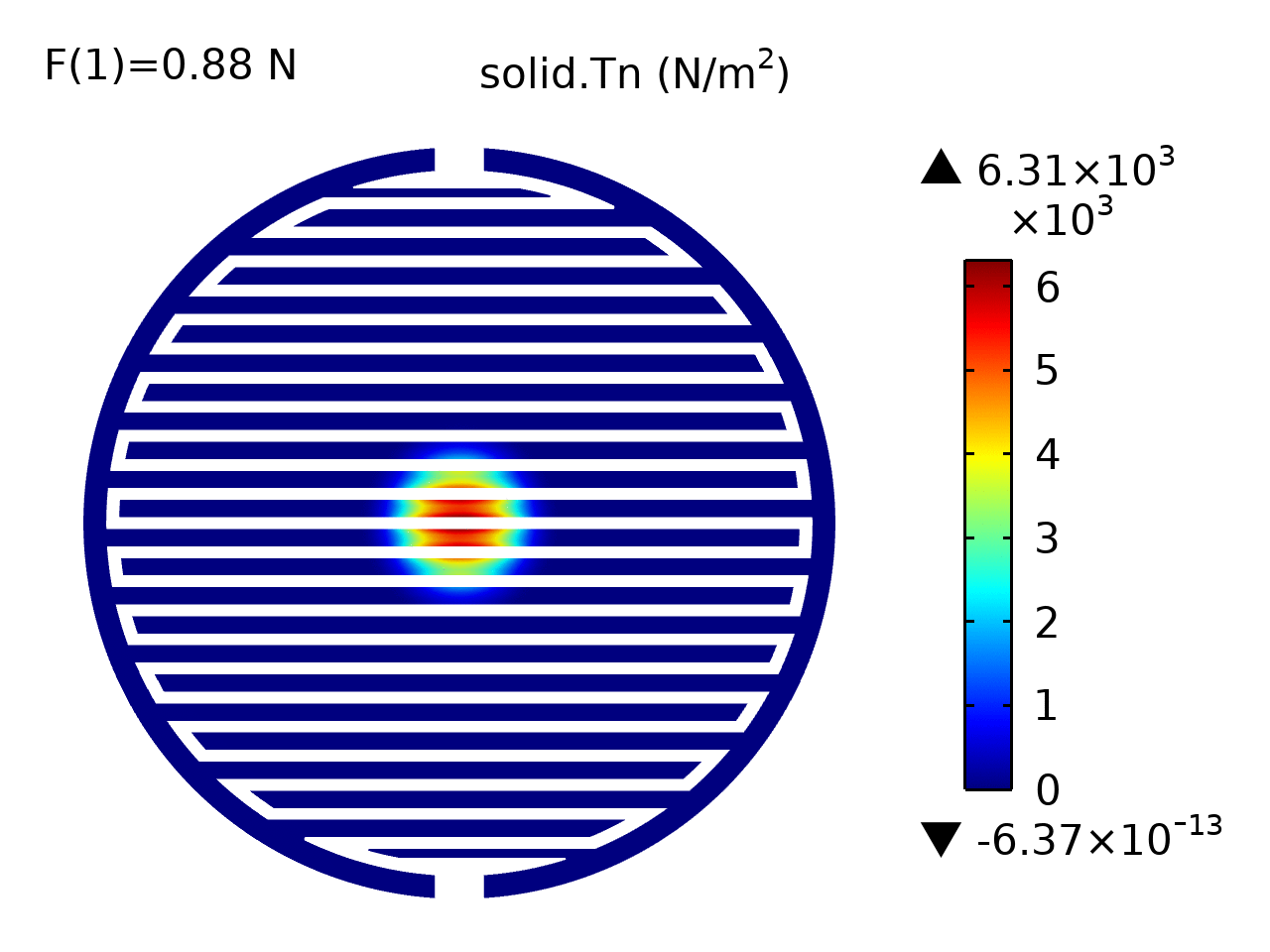

Supplement: Supplementary file 2 — Supplementary Movie S2 [file 41378_2024_717_MOESM2_ESM.gif]

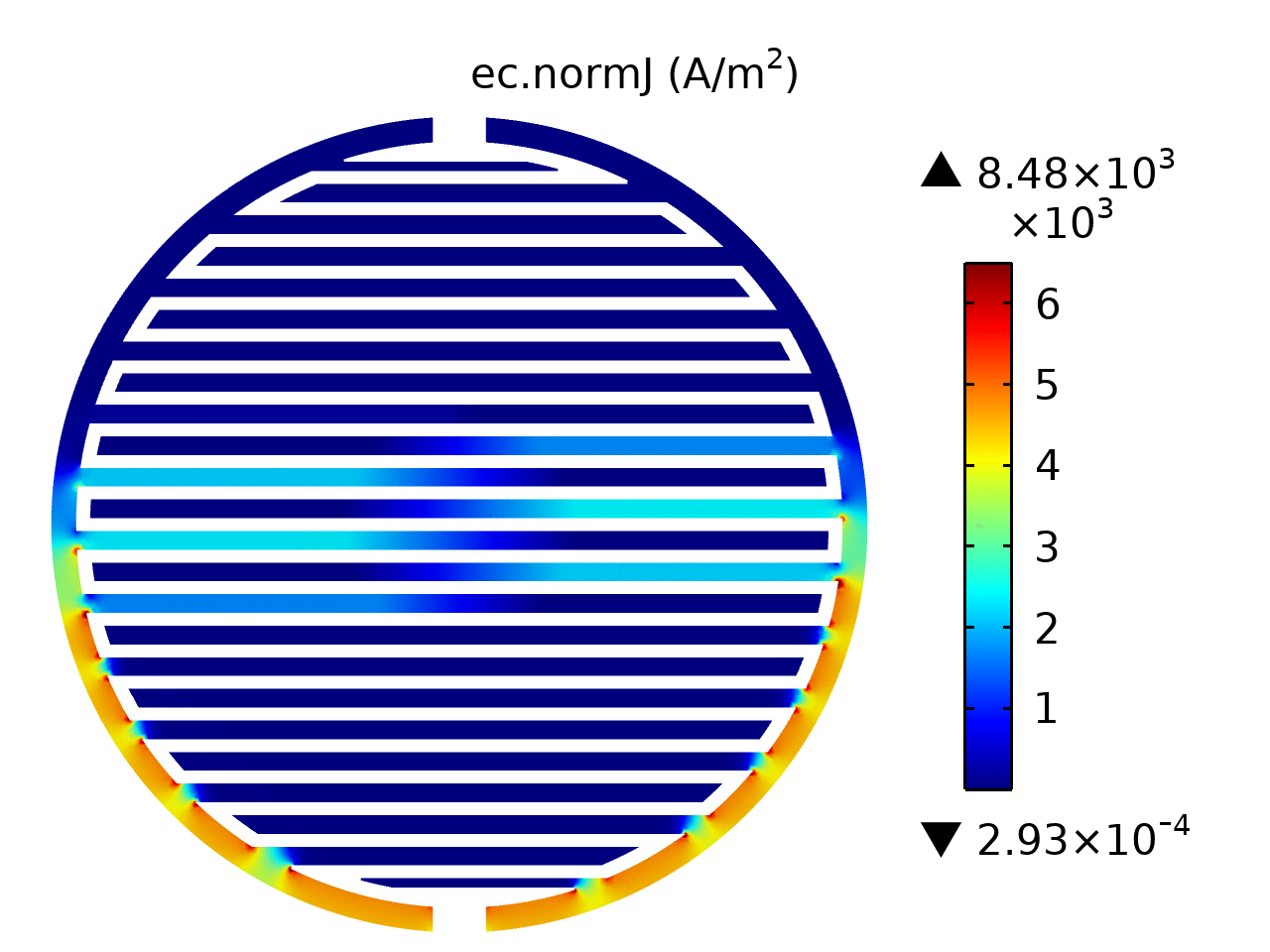

Supplement: Supplementary file 3 — Supplementary Movie S3 [file 41378_2024_717_MOESM3_ESM.gif]
