# Supplementary material for: Rational design of a laminate-structured flexible sensor for human dynamic plantar pressure monitoring
Source: Microsyst Nanoeng. 2024 Jul 16;10:98. doi: 10.1038/s41378-024-00717-1 (PMC11251139; doi:10.1038/s41378-024-00717-1)
Supplement: Supplementary file 4 — Supporting Information [file 41378_2024_717_MOESM4_ESM.docx]

Supporting Information

**Title: Rational Design of a Laminate-Structured Flexible Sensor for Human Dynamic Plantar Pressure Monitoring**

Main setting conditions of the physical fields of solid mechanics and electric currents in the finite element model of the flexible pressure sensor with laminated structure:

From bottom to top, the layers include a polyethylene terephthalate (PET) substrate, silver electrodes, double-sided adhesive shim, air layer, carbon nanomaterial sensing film, another PET substrate and structural steel pressure block.

In the physical field of solid mechanics, the material model is a linear elastic material model, the boundary of the bottom substrate is fixed, and the other boundaries are free to deform. A pressure (p) is applied to the top surface of the device pass through the steel pressure block.

In the physical field of electric currents, an excitation voltage of 2 V is applied to the input ends of the sensor, and the electrical performacne of the sensor was calculated following a current conversion equation based on Ohm’s Law.

The material parameters are set as follows:

Substrate and adhesive shim: PET material, Young's modulus (Es) = 3.1 GPa, Poisson's ratio ($v_{s}$) = 0.37, density (ρ) = 1.30 kg/m^3^, conductivity (σ) = 1e-8 S/m, relative permittivity ($\varepsilon_{r}$) = 4

Interdigitated electrodes: Ag material, Young's modulus (Es) = 12 GPa, Poisson's ratio ($v_{s}$) = 0.21, density (ρ) = 1.05 kg/m^3^, conductivity (σ) = 1e7 S/m, relative permittivity ($\varepsilon_{r}$) = 1

Sensing layer: Carbon nanomaterial, Young's modulus (Es) = 1.5 GPa, Poisson's ratio ($v_{s}$) = 0.28, density (ρ) = 0.79 kg/m^3^, conductivity (σ) = 350 S/m, relative permittivity ($\varepsilon_{r}$) = 4

Structural steel (only involved in the physical field): Young's modulus (Es) = 200 GPa, Poisson's ratio ($v_{s}$) = 0.30, density (ρ) = 7.85 kg/m^3^.

The geometric parameters are set as follows:

Substrate: Diameter = 30 mm, Thickness = 140 μm

Interdigitated electrodes: Width = 0.53 mm, Gap = 0.36 mm, Thickness = 5 μm

Adhesive shim: Width = 2.5 mm, Thickness = 130 μm

Sensing film: Diameter = 25 mm, Thickness = 5 μm

Steel pressure block: Diameter = 15 mm, Height = 5 mm


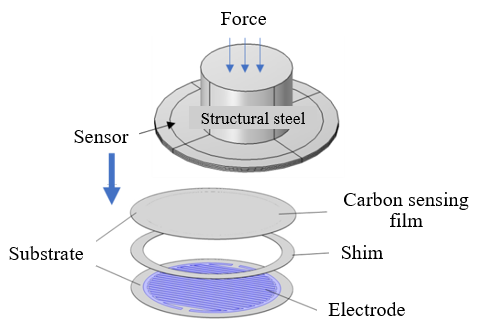


**Figure S1** The finite element model schematic of the flexible pressure sensor with laminate structure


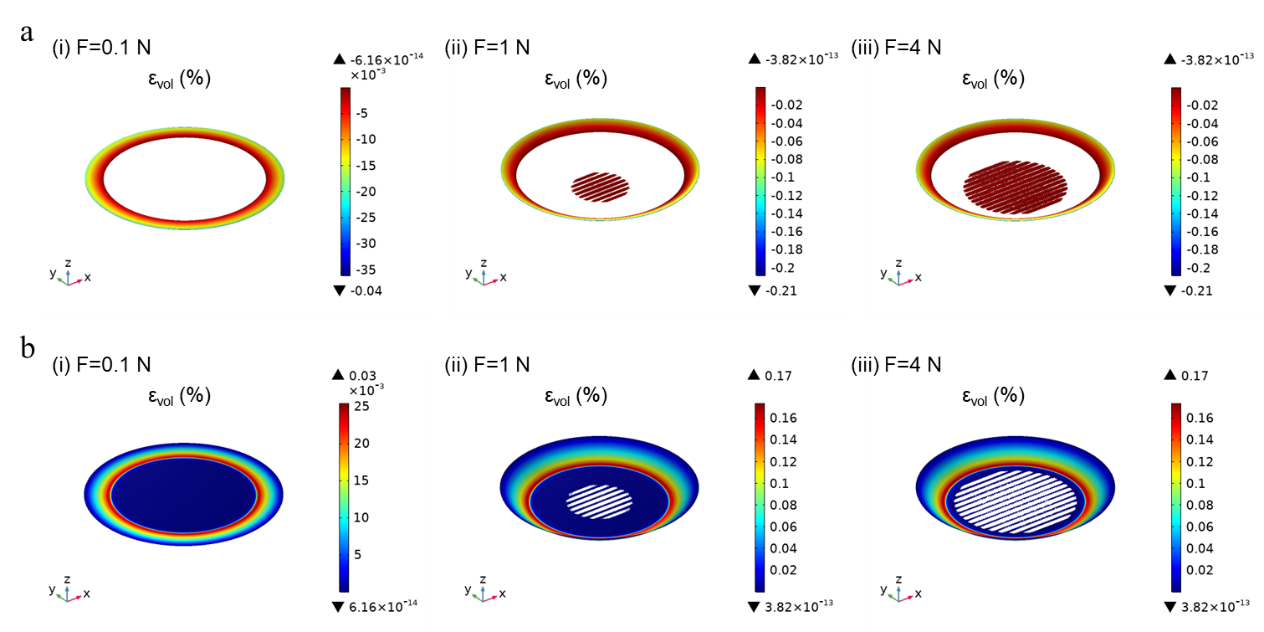


**Figure S2** The volumetric strain (*ε*_vol_) of the sensitive film under different applied force: (a) The compressed area (*ε*_vol_<0); (a) The stretched area (*ε*_vol_>0).


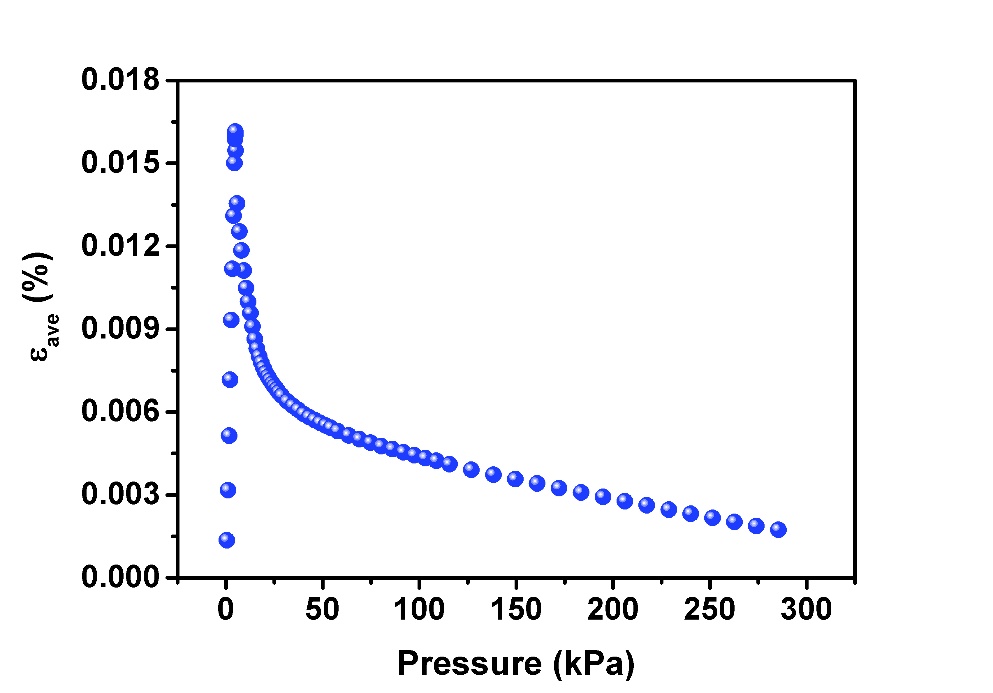


**Figure S3** The average volumetric strain (*ε*_ave_) of the sensitive film as a function of the applied pressure.


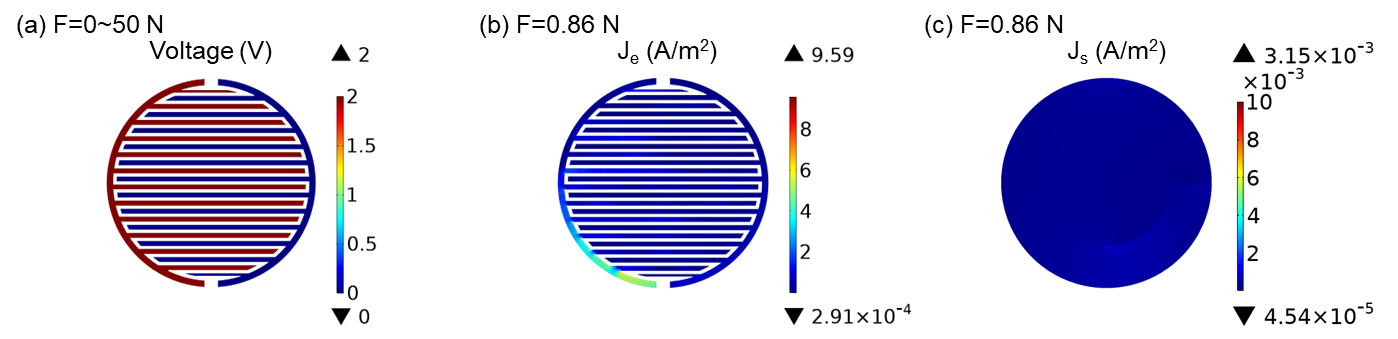


**Figure S4** (a) Voltage distribution on the surface of the interdigital electrodes; (b) Distribution of the current density on the electrode (|J_e_|) and (c) the sensitive layer (|J_s_|) when the sensitive layer and electrode layer are not in contact (F<0.87 N).


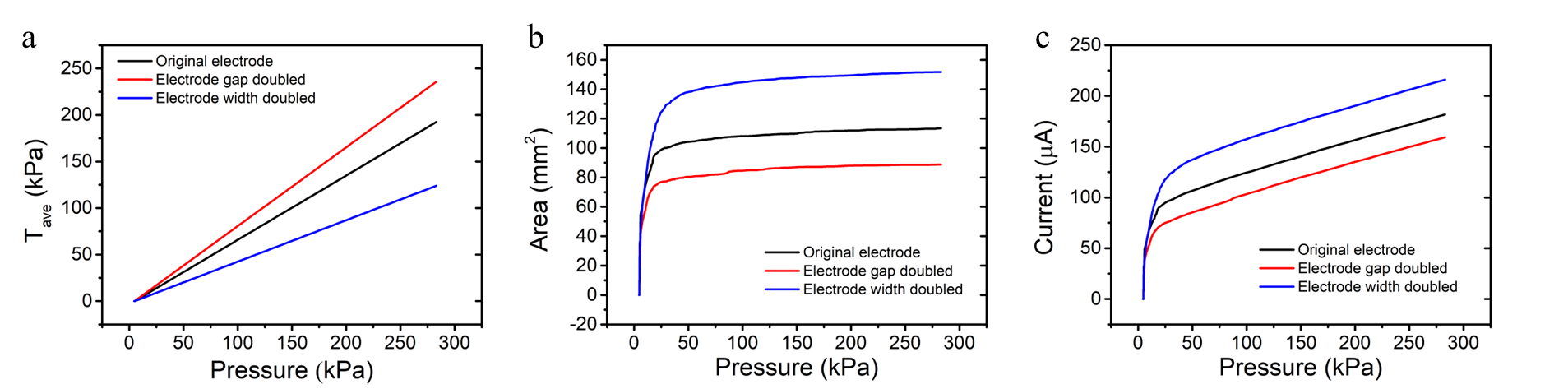


**Figure S5** Influences of the geometric design of the electrodes on (a) the average contact pressure on the electrodes, (b) the contact area, and (c) the current flowing through the flexible pressure sensor.

**
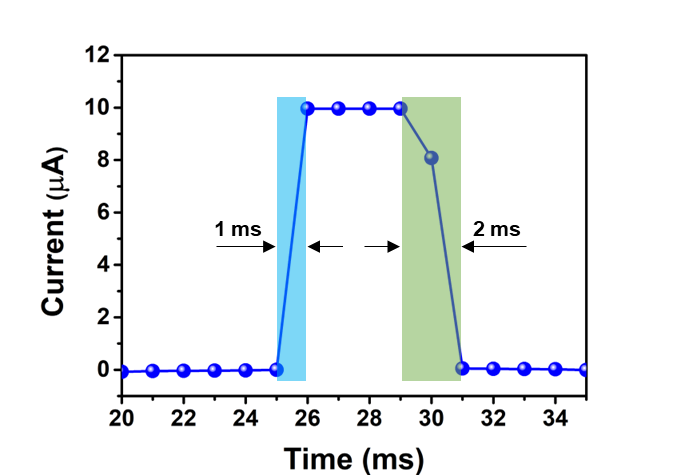
**

**Figure S6** Real-time response of the flexible pressure sensor to pressure showing the response time and the relaxation time.


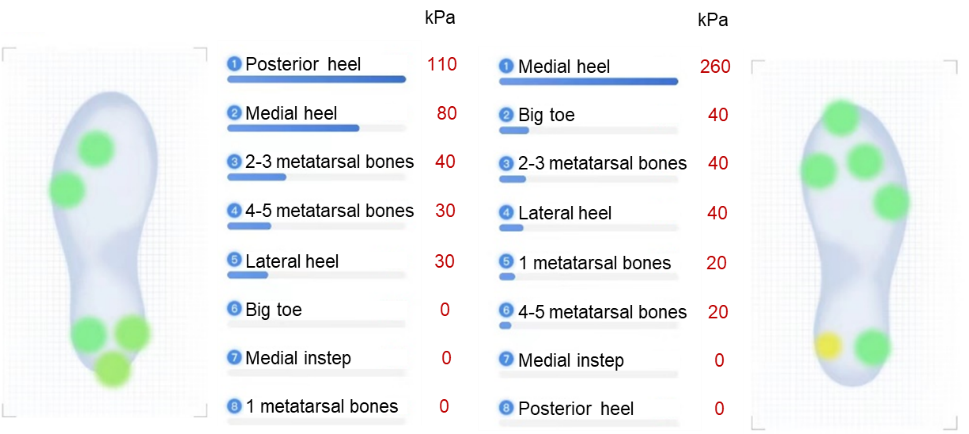


**Figure S7** Pressure values on different parts of the plantars under up-right standing conditions of the human body.

**Table S1** Fitting parameters of Figure 3f

|  | t=60 μm | t=80 μm | t=100 μm | t=120 μm | t=140 μm | t=160 μm | t=180 μm | t=200 μm |
| --- | --- | --- | --- | --- | --- | --- | --- | --- |
| **y0** | -0.22644 | -0.56403 | -0.56403 | -2.08431 | -1.27014 | -3.55689 | -5.89817 | -9.47271 |
| A | 0.19616 | 0.45728 | 0.45728 | 2.16872 | 1.28062 | 3.68971 | 6.09586 | 9.7407 |
| B | 0.01404 | 0.01207 | 0.01207 | 0.009 | 0.01015 | 0.00783 | 0.00677 | 0.00586 |

* Fitting equation: y= y0+ A·exp(B·t)

**Table S2** Fitting parameters of Figure 3g

|  | d=60 μm | d=80 μm | d=100 μm | d=120 μm | d=140 μm | d=160 μm | d=180 μm | d=200 μm |
| --- | --- | --- | --- | --- | --- | --- | --- | --- |
| y0' | -0.79681 | -1.08697 | -1.47187 | -1.98802 | -2.68984 | -3.66115 | -5.03532 | -0.57628 |
| A’ | 0.5128 | 0.76324 | 1.11436 | 1.60948 | 2.31344 | 3.32547 | 4.80182 | 0.33353 |
| B’ | 0.01301 | 0.01242 | 0.01176 | 0.01105 | 0.01029 | 0.00949 | 0.00864 | 0.01351 |

* Fitting equation: y’= y0’+ A’·exp(B’·t)

**Table S3** Comparison of the wearable system in this work to other reported wearable systems for plantar pressure monitoring

| **Sensor Type** | **Detection range** | **Response time** | **Integration way** | **System acquisition frequency** | **System AD conversion resolution** | **System transmission type** | **System analysis function** | **The system output a detection report？** |  |
| --- | --- | --- | --- | --- | --- | --- | --- | --- | --- |
|  |  |  |  |  |  |  | Analyze the proportion of pressure on Plantar？ |  |  |
| Piezoresistive | 300 kPa | 2 ms | Integrated type | 100 Hz | 12 digits | Wireless | Yes | Yes | This Work |
| Piezoresistive | - | - | Splicing type | - | - | Wired | No | No | Ref 1 |
| Capacitive | 200 kPa | 132 ms | Integrated type | 28 Hz | - | Wireless | No | Yes | Ref 2 |
| Triboelectricity | 169 kPa | 12.7 ms | Integrated type | 1000 Hz | - | Wired | No | Yes | Ref 3 |
| Hydrogel | 209 kPa | 69 ms | Integrated type | - | 8 digits | Wireless | No | No | Ref 4 |
| Piezoresistive | - | - | Splicing type | 25 Hz | 8 digits | Wireless | No | No | Ref 5 |
| Capacitive | - | 100 ms | Integrated type | 100 Hz | - | Wireless | Yes | No | Ref 6 |
| Piezoresistive | 700 kPa | - | Splicing type | - | - | Wireless | No | No | Ref 7 |
| Piezoresistive | - | - | Splicing type | 100 Hz | - | Wired | No | No | Ref 8 |
| Piezoresistive | 350 kPa | - | Splicing type | 78 Hz | 10 digits | Wireless | No | No | Ref 9 |
| Piezoresistive | - | - | Splicing type | 6 Hz | 12 digits | Wireless | No | No | Ref 10 |
| Piezoresistive | 250 kPa | - | Splicing type | 20 Hz | 10 digits | Wireless | No | No | Ref 11 |
| - | - | 10 ms | Splicing type | 100 Hz | - | Wireless | No | No | Ref 12 |
| Capacitive | 280 kPa | 200 ms | Integrated type | 100 Hz | - | Wireless | No | No | Ref 13 |
| Photoelectric | 500 kPa | - | Splicing type | 100 Hz | 14 digits | Wireless | No | No | Ref 14 |

References

[1] Swathy, C. R. et al. Wearable foot pressure and gait analysis system. IEEE, DOI: 10.1109/INDICON56171.2022.10040182 (2022).

[2] Tao, J. et al. Real-time pressure mapping smart insole system based on a controllable vertical pore dielectric layer. Microsyst. & Nanoeng. 6, 62 (2020).

[3] Zheng, Q. et al. Self-powered high-resolution smart insole system for plantar pressure mapping. BMEMat 1, e12008 (2023).

[4] Liu, W. et al. Plantar pressure detection system based on flexible hydrogel sensor array and WT-RF. Sensors 21, 5964 (2021).

[5] Chen, J. et al. Plantar pressure‐based insole gait monitoring techniques for diseases monitoring and analysis: a review. Adv. Mater. Technol. 7, 2100566 (2021).

[6] Wang, C. et al. Preliminary clinical application of textile insole sensor for hemiparetic gait pattern analysis. Sensors 19, 3950 (2019).

[7] Muzaffar, S. et al. Piezoresistive sensor array design for shoe-integrated continuous body weight and gait measurement. IEEE,

DOI: 10.1109/DTIP.2019.8752629 (2019).

[8] Healy, A. et al. Repeatability of walkinSense® in shoe pressure measurement system: a preliminary study. The Foot 22, 35–39 (2012).

[9] Razak, A. H. A. et al. Foot plantar pressure measurement system: a review. Sensors 12, 9884-9912 (2012).

[10] Benocci, M. et al. A wireless system for gait and posture analysis based on pressure insoles and inertial measurement units. 3d International ICST Conference on Pervasive Computing Technologies for Healthcare, DOI: 10.4108/ICST.PERVASIVEHEALTH2009.6032 (2009).

[11] Saito, M. et al. An in-shoe device to measure plantar pressure during daily human activity. Med. Eng. Phys. 33, 638–645 (2011).

[12] Lee, S. et al. Parkinson’s disease classification using gait characteristics and wavelet-based feature extraction. Expert Systems with Applications 39, 7338-7344 (2012).

[13] Feger, M. A. et al. Surface electromyography and plantar pressure changes with novel gait training device in participants with chronic ankle instability. Clin. Biomech. 37, 117-124 (2016).

[14] Rossi, S. et al. Development of an in-shoe pressure-sensitive device for gait analysis. IEEE Engineering in Medicine and Biology Society, DOI:10.1109/IEMBS.2011.6091364 (2011).

**Supplementary Movie Captions**

**Movie** S**1.** The variation of the mechanical deformation and stress distribution of the sensor under different forces.

**Movie** S**2.** The variation of the contact pressure at the interface between the sensitive layer and the electrode layer of the sensor under different forces.

**Movie** S**3.** The synchronous changes of the current density in the electrode layer when the applied force increases.
